# Identification of the Begomoviruses Squash Leaf Curl Virus and Watermelon Chlorotic Stunt Virus in Various Plant Samples in North America

**DOI:** 10.3390/v13050810

**Published:** 2021-04-30

**Authors:** Rafaela S. Fontenele, Amulya Bhaskara, Ilaria N. Cobb, Lucas C. Majure, Andrew M. Salywon, Jesús A. Avalos-Calleros, Gerardo R. Argüello-Astorga, Kara Schmidlin, Philippe Roumagnac, Simone G. Ribeiro, Simona Kraberger, Darren P. Martin, Pierre Lefeuvre, Arvind Varsani

**Affiliations:** 1 The Biodesign Center for Fundamental and Applied Microbiomics, Arizona State University, Tempe, AZ 85287, USA; rafasfontenele@asu.edu (R.S.F.); amulyabhaskara@gmail.com (A.B.); ilaria.cobb@gmail.com (I.N.C.); kara.schmidlin@asu.edu (K.S.); simona.kraberger@asu.edu (S.K.); 2 School of Life Sciences, Arizona State University, Tempe, AZ 85287, USA; 3 School of Behavioral and Brain Sciences, University of Texas at Dallas, 800 W Campbell Rd, Richardson, TX 75080, USA; 4 The University of British Columbia, Vancouver, BC V6T 1Z4, Canada; 5 Florida Museum of Natural History, University of Florida, Gainesville, FL 32611, USA; lmajure@floridamuseum.ufl.edu; 6 Desert Botanical Garden, Phoenix, AZ 85008, USA; asalywon@dbg.org; 7 División de Biología Molecular, Instituto Potosino de Investigación Científica y Tecnológica, A.C., Camino a la Presa de San José 2055, Lomas 4ta Secc, San Luis Potosi 78216, Mexico; jesus.avalos@ipicyt.edu.mx (J.A.A.-C.); grarguel@ipicyt.edu.mx (G.R.A.-A.); 8 CIRAD, UMR PHIM, 34090 Montpellier, France; philippe.roumagnac@cirad.fr; 9 PHIM Plant Health Institute, Univ Montpellier, CIRAD, INRAE, Institut Agro, IRD, 34090 Montpellier, France; 10 Embrapa Recursos Genéticos e Biotecnologia, Brasília 70770-917, Brazil; simone.ribeiro@embrapa.br; 11 Computational Biology Division, Department of Integrative Biomedical Sciences, Institute of Infectious Diseases and Molecular Medicine, University of Cape Town, Observatory, 7925 Cape Town, South Africa; darrenpatrickmartin@gmail.com; 12 CIRAD, UMR PVBMT, F-97410 St. Pierre, France; pierre.lefeuvre@cirad.fr; 13 Center for Evolution and Medicine, Arizona State University, Tempe, AZ 85287, USA; 14 Structural Biology Research Unit, Department of Integrative Biomedical Sciences, University of Cape Town, 7925 Cape Town, South Africa

**Keywords:** *Geminiviridae*, *begomovirus*, pseudo-recombination, recombination

## Abstract

Geminiviruses are a group of plant-infecting viruses with single-stranded DNA genomes. Within this family, viruses in the genus *Begomovirus* are known to have a worldwide distribution causing a range of severe diseases in a multitude of dicotyledonous plant species. Begomoviruses are transmitted by the whitefly *Bemisia tabaci,* and their ssDNA genomes can be either monopartite or bipartite. As part of a viral survey, various plants including those in the families Alliaceae, Amaranthaceae, Apiaceae, Asteraceae, Brassicaceae, Cactaceae, Cucurbitaceae, Lamiaceae, Lauraceae, Malvaceae, Oleaceae and Solanaceae were sampled and screened for begomoviruses using both a high-throughput sequencing and a begomovirus-specific primer pair approach. Based on the sequences derived using these approaches, the full-length genome of various begomoviruses were amplified from plants using abutting primers. Squash leaf curl virus (SLCV) and watermelon chlorotic stunt virus (WCSV) were identified in Cactaceae (*n* = 25), Solanaceae (*n* = 7), Cucurbitaceae (*n* = 2) and Lamiaceae (*n* = 1) samples. WCSV is an Old World bipartite begomovirus that has only recently been discovered infecting watermelons in the Americas. Our discovery of WCSV in the USA is the first indication that it has reached this country and indicates that this virus might be widespread throughout North America. Phylogenetic analysis suggests WCSV was introduced to the New World twice. The detection of begomoviruses in cactus plants suggests possible spillover events from agricultural areas into native vegetation. Since WCSV and SLCV have previously been found in mixed infections, pseudo-recombination infection experiments were conducted. We demonstrate that WCSV DNA-B is successfully trans-replicated by SLCV DNA-A despite very low degree of similarity between the replication-associated iterative sequences present in their common region, an essential feature for binding of the replication associated protein. This study highlights the importance of viral surveys for the detection of spillover events into native vegetation, but also suggests the need for more surveillance of WCSV in the USA, as this virus is a serious threat to watermelon cultivation in the Middle East.

## 1. Introduction

The family *Geminiviridae* is a group of plant-infecting viruses with circular single-stranded DNA genomes encapsidated in twinned semi-icosahedral particles [1]. Within this family, the members of the genus *Begomovirus* have a worldwide distribution, infect dicotyledonous plants and are transmitted by whiteflies (*Bemisia tabaci*). With over 424 species, the genus *Begomovirus* is the largest geminivirus genus. Begomoviruses have emerged as severe pathogens causing high yield losses on a variety of economically important crops [2,3]. Begomovirus genomes have either one (monopartite) or two (bipartite) genomic components named DNA-A and DNA-B. Monopartite begomoviruses are mainly found in the Old World, whereas New World begomoviruses are predominantly bipartite. In bipartite begomoviruses, the DNA-A component encodes five proteins, the replication associated protein (Rep), replication enhancer protein (Ren), transactivator protein (Trap), a capsid protein (CP) and C4, a symptom determinant protein. The DNA-B encodes a movement protein (MP) and a nuclear shuttle protein (NSP); hence, the DNA-B component is responsible for cell-to-cell and long distance movement within the infected plant. For bipartite begomoviruses, cognate DNA-A and DNA-B components share a common region (CR) in the intergenic region, having a high-enough degree of similarity to allow the Rep encoded by DNA-A to replicate both components.

Squash leaf curl virus (SLCV) is a typical New World bipartite begomovirus that was initially identified and characterized infecting squash in the USA [4,5] and since then has been shown to be widespread throughout North America [6,7,8]. In 2002, SLCV was identified infecting squash plants in Israel [9], which was the first time a New World begomovirus was documented in the Old World. SLCV is now widely spread in the greater Middle East (Egypt, Jordan, Lebanon, Oman, Pakistan and Palestine) [10,11,12,13,14,15]. Although SLCV mainly infects squash plants, causing severe curling and yellow mottling of leaves, the host range of this begomovirus includes all major cultivated crops from the family Cucurbitaceae. In addition, it has also been identified infecting some Solanaceae species including tomato and pepper, two Malvaceae species (cotton and *Malva parviflora*) and an uncultivated Brassicaceae species (*Sinapis arvensis*). SLCV is also commonly found in mixed infections with tomato yellow leaf curl virus, cucurbit leaf crumple virus and watermelon chlorotic stunt virus, mixtures which usually increase disease severity [7,16,17].

Watermelon chlorotic stunt virus (WCSV) is a bipartite begomovirus of the Old Word that was initially identified infecting watermelon in Yemen [18] but has been extensively described in several Middle East countries/regions (Iran, Israel, Jordan, Lebanon, Oman, West bank, Palestine and Saudi Arabia) and Sudan in Africa [15,19,20,21,22,23,24,25]. WCSV can also infect other cucurbits but mainly causes severe damage to watermelon crops [15]. Recently, WCSV has been found infecting watermelon in Sonora, Mexico [26] causing the typical symptoms of leaf curling and yellowing, which was the first identification of this begomovirus species in the Western hemisphere (i.e., New World).

Here, we describe and characterize isolates of SLCV and WCSV found infecting plants in the Cactaceae, Cucurbitaceae, Lamiaceae and Solanaceae families in the USA and Mexico. The three WCSV isolates reported in this study are the first identification of this virus in the USA, and their presence shows that this virus is likely more widespread in North America than previously known. The identification of both SLCV and WCSV infecting Cactaceae species provides evidence of spillover events from agro-ecosystems into native vegetation. The biological implications of begomoviruses infecting cactus plants need to be further investigated. The demonstration that a cactus-derived SLCV DNA-A was able to replicate a cactus-derived WCSV DNA-B in *Nicotiana benthamiana* plants and cause mild curling symptoms suggests that these invasive pathogens may pose new ecological and/or agricultural threats.

## 2. Materials and Methods

### 2.1. Sample Collection and Processing

Cactaceae plants were sampled as part of a viral survey and processed as described in [27]. In addition, Alliaceae, Amaranthaceae, Apiaceae, Asteraceae, Brassicaceae, Cucurbitaceae, Lamiaceae, Lauraceae, Malvaceae, Oleaceae and Solanaceae plants were sampled from five different locations in Arizona (USA); a community garden (*n* = 3); three farms (*n* = 20) and an herb garden at the Desert Botanical Garden in Phoenix, AZ (*n* = 25) (Supplementary Table S1). Total DNA was extracted from non-cactus samples using either the GeneJET Plant Genomic DNA Purification Kit (Thermo Fisher Scientific, Waltham, MA, USA) or the GenCatch Plant Genomic DNA Purification Kit (Epoch Life Science, Missouri City, TX, USA) according to the manufacturer’s instructions.

### 2.2. Genome Assembly and Annotation

Using previously acquired high-throughput sequencing data from cactus samples [27], the contigs were mined for those with similarities to begomoviruses. Pairs of abutting primers were designed to recover the full-length DNA-A and DNA-B components of watermelon chlorotic stunt virus (WCSV) and squash leaf curl virus (SLCV) from cactus and other sampled plants (Supplementary Table S1). Samples were also screened with the universal primer pair PAL1v1978/PAR1c715 for the DNA-A component of begomoviruses [28] to identify begomovirus positive samples, these amplicons were cloned and sequenced to confirm their identity.

PCR with primers listed in Supplementary Table S2 (designed based on de novo assembled high throughput sequencing data) was preformed using KAPA HiFi HotStart DNA polymerase (KAPA Biosystems, Wilmington, MA, USA). Amplicons were resolved in a 0.7% agarose gel and amplicon with a size of ~2.5–3 kb were excised, gel purified and cloned in the pJET1.2 cloning vector (Thermo Fisher Scientific, Waltham, MA, USA). The recombinant plasmids were Sanger sequenced by primer walking at Macrogen Inc. (Seoul, South Korea), and assembly and annotations were carried out using Geneious 11.1.5 (Biomatters Ltd., Auckland, New Zealand).

### 2.3. Infectivity Assays

Infectivity assays were performed with isolates of DNA-A and DNA-B from both WCSV and SLCV. For the WCSV, the DNA-A component isolate LCM_52 (GenBank accession MW588390) and the DNA-B component isolate LCM_53 (GenBank accession MW588417) were used to generate two infectious clones. For the infectious clones of the SLCV, the DNA-A component isolate LCM_89_SP41 (GenBank accession MW588381) and the DNA-B isolate LCM_89_SP58 (GenBank accession MW588407) were used. These DNA-A and DNA-B components were recovered from cactus samples (Table 1). Specific primers were designed to amplify two copies of each cactus-derived begomoviruses component that were cloned in tandem to the binary vector pJL-89 [29], with the 35S promoter excluded as previously described by Ferro et al. [30], using Gibson assembly [31] (New England Biolabs, Ipswich, MA, USA). Each clone was transformed into competent *Escherichia coli* XL1 Blue cells. To further confirm the correct orientations of the cloned tandem genomes, clones were analyzed by restriction enzyme digestion: SLCV DNA-A and DNA-B, WCSV DNA-B using EcoRV and WSCV DNA-A using SalI. Clones containing the two tandemly cloned copies of DNA-A or DNA-B from SLVC or WCSV were used to transform *Rhizobium radiobacter* GV3101.

Infection assays were performed in *Nicotiana benthamiana* plants. In each experiment ten *N. benthamiana* plants were inoculated with WCSV DNA-A/DNA-B or SLCV DNA-A/DNA-B. To investigate whether the DNA-B of each virus could be efficiently replicated by the DNA-A of the other, infection assays were also performed with either SLCV DNA-A/WCSV DNA-B and WCSV DNA-A/SLCV DNA-B combinations. In all inoculations, *R. radiobacter* was grown for 20 h in Luria broth with kanamycin (50 µg/mL) and rifampicin (50 µg/mL). The culture was then centrifuged for 10 min at 4600 rpm to pellet the cells before resuspension in MES buffer (10 mM MES hydrate and 10 mM MgSO4•7H_2_O) with 150 µM of acetosyringone to an OD_600nm_ of 1.0. Equal volumes of each component (OD_600nm_ 1.0) were mixed together prior to inoculation. Systemic infection was tested by collecting newly emerged leaves. Total DNA extractions from leaf material were performed using the GenCatch Plant Genomic DNA Purification Kit (Epoch Life Science, Missouri City, TX, USA) according to the manufacturer’s instructions. Total DNA was tested by PCR with pairs of abutting primers for each virus component (Supplementary Table S2) using KAPA HiFi HotStart DNA polymerase (KAPA Biosystems, Wilmington, MA, USA).

### 2.4. Sequence Analyses

The full-length sequence of each component from the SLCV and WCSV together with available genomes from the same species (downloaded from GenBank on 16 December 2020) were used to generate a dataset for each virus component. For each dataset of SCLV and WCSV components, sequences were aligned using MAFFT v.7 [32] and subsequently used to detect recombination using the program RDP v.5.5 [33]. The methods RDP [34], GENECONV [35], BOOTSCAN [36], MAXCHI [37], CHIMERA [38], SISCAN [39] and 3SEQ [40] were used with default parameters for the recombination analysis. Only recombination events that were detected by more than three methods with a *p*-value <0.05 were accepted.

Alignments with recombinant regions removed were used for phylogenetic analysis. Following ModelFinder [41] analyses, the nucleotide substitution models used were TIM + F+G4, TIM3 + F+G4, TIM2 + F+G4 and HKY + F+G4 for WCSV DNA-A, WCSV DNA-B, SLCV DNA-A and SLCV DNA-B, respectively. The Maximum-Likelihood (ML) phylogenetic tree for each dataset was inferred using IQ-TREE [42] with 1000 bootstrap replicates for branch support from which branches with <60% support were collapsed using TreeGraph2 [43]. With the exception of the SLCV DNA-A ML phylogenetic tree that was rooted with SLCV DNA-A sequences from Pakistan (MF504011 and MF504010); all other ML phylogenetic trees were rooted with other begomovirus sequences. The ML phylogenetic trees were visualized and edited using iTOL [44]. All pairwise identities were determined using SDTv1.2 [45].

## 3. Results and Discussion

After a viral survey on cactus samples using high-throughput sequencing [27], 23 contigs with similarities to WCSV and SLCV were identified. Amplification and cloning of possible full-length genomes using abutting primers designed from these contigs showed the presence of the begomovirus squash leaf curl virus (SLCV) and watermelon chlorotic stunt virus (WCSV) in 25 cactus plants. Additional non-cactus plants sampled from five locations in the state of Arizona were also screened for the presence of SLCV and WCSV, and from those, 10 plants were found to be infected. In some cases, only one component from each virus could be detected (Table 1). In samples where only DNA-B could be identified, no other begomovirus was detected using the degenerate begomoviruses DNA-A component primer pair, PAL1v1978/PAR1c715 [28]. However, for the *Opuntia robusta* (Lab ID DBG34) sample in which the SLCV DNA-B was identified, Opuntia virus 1 had already been characterized through high throughput sequencing based approaches [27]. In addition, two other samples (Table 1) that only contained DNA-B components also had sub-genomic molecules with similarity to becurtoviruses and Opuntia virus 1. Therefore, it is highly likely for the plant where only the DNA-B was identified, that other geminiviruses were present but we were unable to detect them. Importantly, these might be able to replicate the DNA-B component.

### 3.1. Squash Leaf Curl Virus

A total of 24 SLCV DNA-A and 22 SLCV DNA-B component sequences from 29 plants including cactus (Cactaceae) (*n* = 21), eggplant (Solanaceae) (*n* = 1), tomato (Solanaceae) (*n* = 3), zucchini (Cucurbitaceae) (*n* = 1), watermelon (Cucurbitaceae) (*n* = 1) and pepper (Solanaceae) (*n* = 2) (Table 1) were determined. Both SLCV DNA-A and DNA-B were identified in 10 samples, and only DNA-A was identified and recovered from nine samples and only DNA-B from 10 samples (Table 1). To our knowledge, SLCV had been previously identified in plants from the families Brassicaceae, Cucurbitaceae, Malvaceae and Solanaceae but never from Cactaceae. From the 21 cactus plants analyzed, four presented both DNA-A and DNA-B, eight presented only DNA-A and the other nine samples only DNA-B.

The SLCV DNA-A component sequences share 92.5–98.3% pairwise identity with other SLCV DNA-A isolates and 98.6–100% identity amongst themselves (Supplementary Data S1). The SLCV DNA-B component sequences share 88.7–95.3% pairwise identity with other SLCV DNA-B isolates and 94.5–100% identity amongst themselves (Supplementary Data S1).

**Figure 1:**
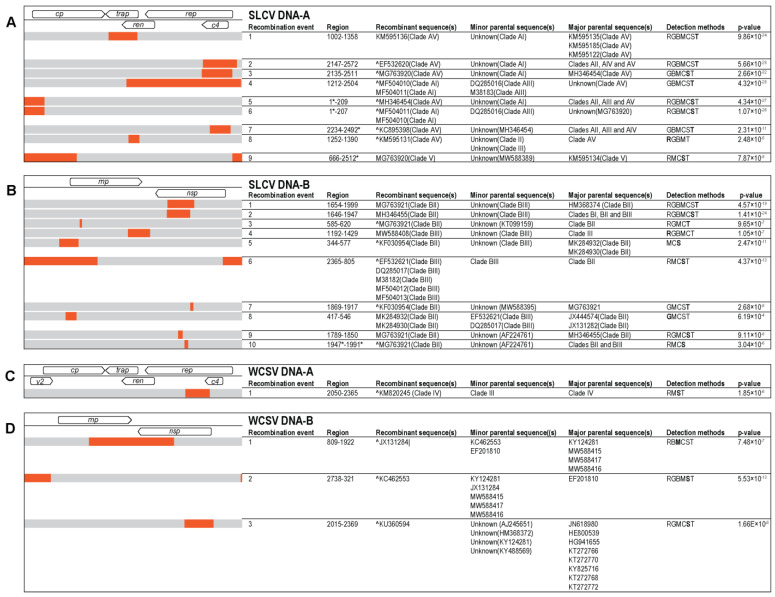
Summary of the recombination events detected in the datasets of (**A**). SLCV DNA-A, (**B**). SLCV DNA-B, (**C**). WCSV DNA-A and (**D**). WCSV DNA-B by RDP5 v.5.5 [33]. The methods used to detect recombination are RDP (R) GENCONV (G), BOOTSCAN (B), MAXCHI (M), CHIMERA (C), SISCAN (S) and 3SEQ (T). The method with the highest *p*-value for each recombination event is in bold text. Sites where the actual breakpoint is undetermined are marked with *. Recombinant sequences marked with ^ indicated that recombinant sequence may have been misidentified (one of the identified parents might be the recombinant). On the right hand side of the table there is a graphical representation of each genome. Each recombination event is represented in orange relative to the reference genome.

Recombination analysis of the SLCV DNA-A and DNA-B sequences from this study and others available in GenBank resulted in the identification of nine putative recombination events for DNA-A and 10 for DNA-B (Figure 1A,B). In the DNA-A dataset (*n* = 162), eight sequences were found to be recombinants, one of which presented two recombination events (MG763920). The recombinantionally derived genomic fragments ranged in size from 138 nts to 1292 nts with the majority occurring in the virion sense genes. For the SLCV DNA-B dataset (*n* = 44) eleven sequences were identified as recombinants with one sequence (MG763921) presenting four recombination events. Recombination event #6 was identified in five sequences and event #4 represents a recombination that occurred between sequences recovered from this study (USA). The transferred fragments ranged from 35 to 1000 nts.

**Figure 2:**
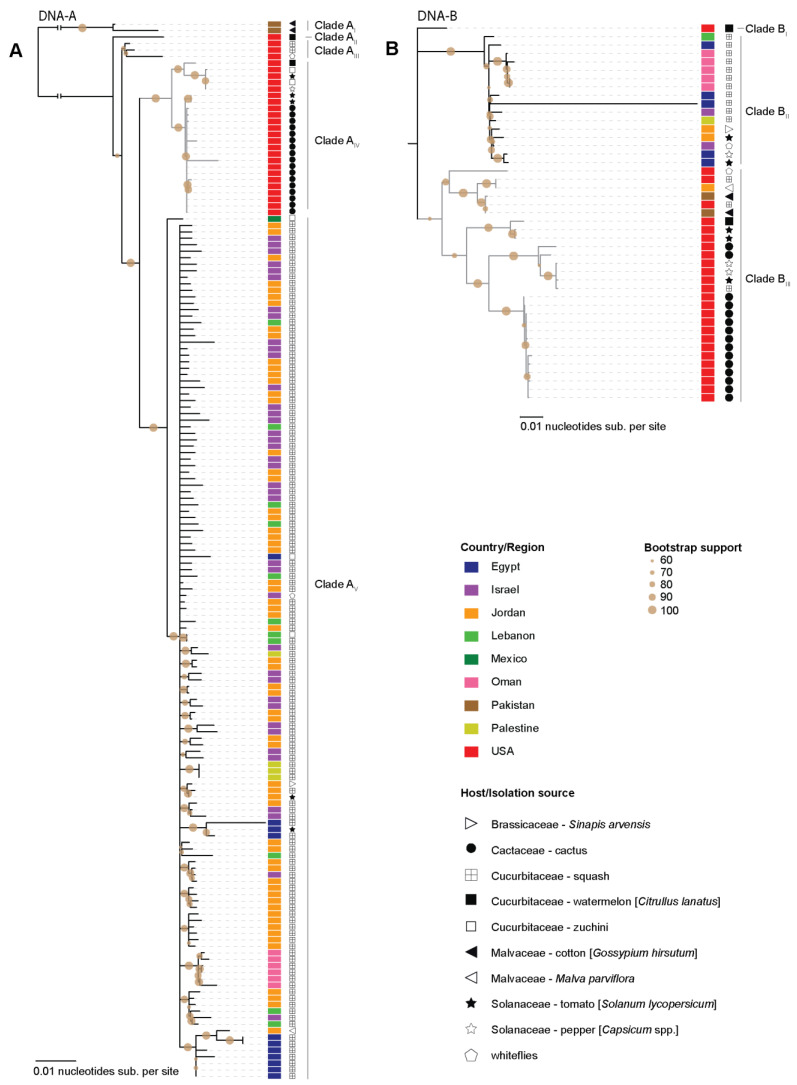
Maximum-Likelihood phylogenetic tree of the SCLV (**A**). DNA-A and (**B**). DNA-B component sequences. The country from which the sequences have been identified are color coded and the host/isolation source is marked by symbols. Clades containing the sequences from this study are colored in grey.

Phylogenetic analysis of the SLCV DNA-A and DNA-B sequences indicated a degree of geographical clustering (Figure 2). The SLCV DNA-A sequences identified in this study all cluster within the same clade (Figure 2A. Clade A_IV_). In this clade all the cactus-derived sequences cluster together and are collectively most closely related to SLCV isolates from tomato plants (Figure 2A. Clade A_IV_). There is a distinct clade containing SLCV DNA-A sequences from the Middle East (Figure 2A. Clade A_V_). Interestingly, there is one SLCV DNA-A sequence recovered from zucchini in Mexico that stands out from this clade (Clade A_V_). Basal to clades A_IV_ and A_V_ are other SLCVs from the USA that infect watermelon and squash (including whiteflies found feeding on squash) (Figure 2A. Clades A_II_ and A_III_) and two more diverse sequences from Pakistan found infecting cotton plants (Figure 2A. Clade A_I_). The geographical clustering observed for the Middle East sequences is similar to that which has been reported previously [46]. Based on the phylogeny it seems like SLCV could have been introduced once into the Middle East. The Pakistan isolates share ~92% pairwise identity with other SLCV isolates. Due to sampling bias, it is difficult to identify any associations between different SLCV clades and the host plants from which they were sampled.

Phylogenetic analysis of the SLCV DNA-B sequences revealed three distinct clades (Figure 2B). Clade B_II_ contains SLCV DNA-B sequences from the Middle East and Clade B_III_ contains all but one of the North American sequences, two sequences from Pakistan and one from Jordan. Lastly, one sequence from the USA identified infecting watermelon [47] sits outside those two clades (Clade B_I_). This pattern suggests that DNA-B may have been introduced to the Middle-East at least twice. These apparently distinct DNA-A and DNA-B evolutionary histories highlight once again the importance of component reassortments for bipartite begomoviruses [48,49].

### 3.2. Watermelon Chlorotic Stunt Virus

Three WCSV DNA-A isolates and three DNA-B isolates were recovered from six plants from the Cactaceae, Lamiaceae and Solanaceae families (Table 1). To our knowledge, with the exception of one other Solanaceae plant (*Datura innoxia*) [50], this is the first identification of WCSV from plants in the Cactaceae and Lamiaceae.

The three WCSV DNA-A sequences share >97.7% genome pairwise identity amongst themselves and 96.5–99.7% identity with other WCSV DNA-A sequences available in GenBank (Supplementary Data S2). The WCSV DNA-B sequences share 92–99% genome pairwise identity with other WCSV DNA-B sequences and 99.9% sequence identity amongst them. Similar to what was observed for the SLCV components, the WCSV DNA-B sequences are more diverse than the WCSV DNA-A sequences.

**Figure 3:**
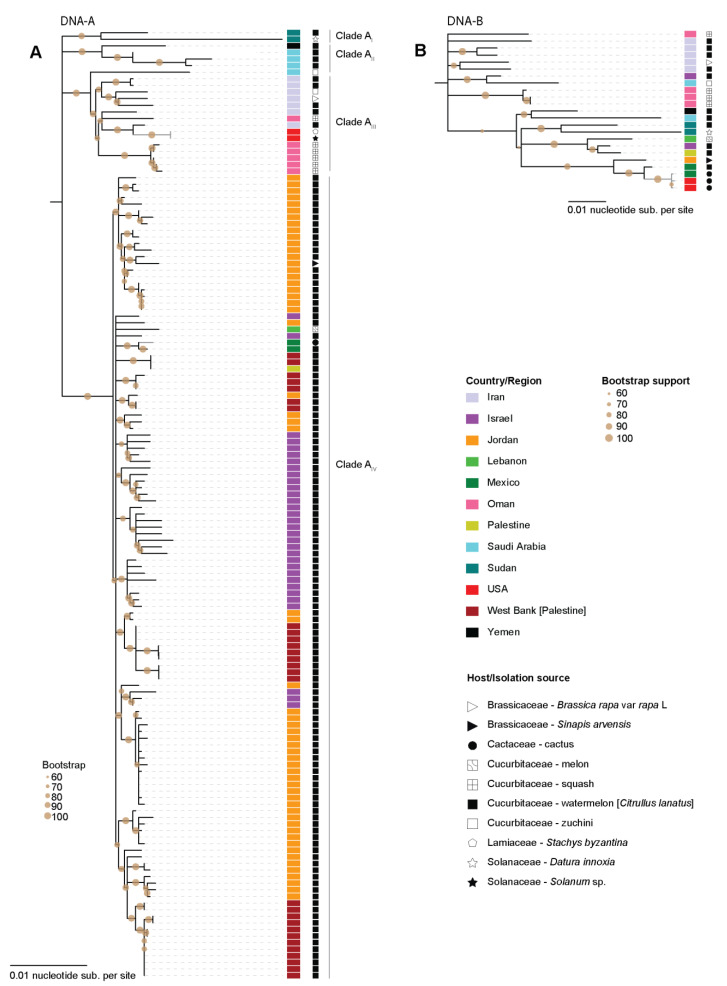
Maximum-Likelihood phylogenetic tree of the WCSV (**A**). DNA-A and (**B**). DNA-B component sequences. The country from which the sequences have been identified are color coded and the host/isolation source is marked by symbols. Clades containing the sequences from this study are colored in grey.

A single putative recombinant region was identified in the WCSV DNA-A dataset (*n* = 144) (Figure 3). The recombinant sequence was isolated from a watermelon plant in Israel. The recombinationally derived genome fragment was 315 nucleotides long and includes a portion of the *rep* and *ac4* coding regions. Meanwhile, three recombinant regions were identified in the WCSV DNA-B dataset (Figure 3). The recombinationally transferred genome fragments ranged in size from 343 nts to 1113 nts (~40% of the genome). No recombination was detected in any of the WCSV sequences recovered in this study.

The ML phylogenetic tree of the WCSV DNA-A sequences has four clades displaying some degree of geographical clustering (Figure 3A). In Clade A_I_, there are two sequences from Sudan while Clade A_II_ includes sequences from Saudi Arabia and Yemen. Clade A_III_ includes a sequence from Saudi Arabia in addition to sequences from Iran and Oman, and two of the three sequences from this study. Clade A_IV_ is the largest and is mainly composed of sequences of isolates from watermelon plants. There are two groups within this clade, one composed mainly of sequences of isolates from Jordan and the other includes those from Israel, Lebanon, Palestine and two from Mexico recovered from watermelon [26] and cactus from this study. Based on this analysis, WCSV DNA-A seems to have been introduced to North America twice. The WCSV DNA-A sequence from cactus sits closely together with the Mexico isolate and those isolates share 99.7% pairwise identity (Supplementary Data S2).

The ML phylogenetic analysis of the WCSV DNA-B does not reveal clear clade structuring but shows similar geographical trends to the DNA-A phylogenetic analysis (Figure 3B). The three WCSV DNA-B sequences recovered from this are closely related to the WCSV DNA-B obtained from watermelon plants from Mexico in 2012, which was the first identification of WCSV in North America [26].

### 3.3. Infectivity Assays for Pseudo-Recombination

WCSV and SLCV have been previously found in mixed infection [20] and in some cases synergistically interact to cause more severe symptoms than either virus alone [15,17]. During mixed infection, begomoviruses can also undergo pseudo-recombination (or reassortment), i.e., DNA-A and DNA-B components from two different viruses form a new association where the DNA-B is successfully replicated by the DNA-A encoded Rep protein. Both WCSV and SLCV have been, respectively, been found to pseudo-recombine (also referred to as reassortment) with the begomoviruses tomato leaf curl Palampur virus and cucurbit leaf curl virus [6,51]. However, to our knowledge pseudo-recombination between WCSV and SLCV have never been found in nature and have never been experimentally tested.

**Figure 4:**
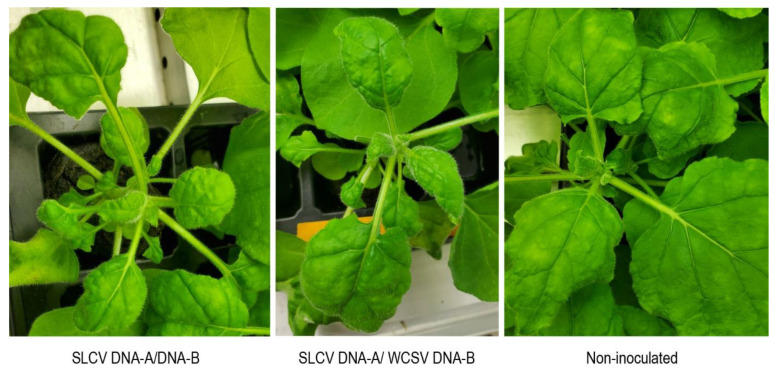
*Nicotiana benthamiana* plants infected with SLCV DNA-A/DNA-B; SLCV DNA-A/WCSV DNA-B showing leaf curling symptoms. As a control, an image of a noninoculated *N. benthamiana* plant.

Infectivity assays with a combination of DNA-A and DNA-B components from both viruses were conducted. For the control experiment with both components of SLCV 10/10, *N. benthamiana* plants were systemically infected 15 days post inoculation as determined by PCR on total DNA of newly emerged leaves. The infected plants displayed symptoms of leaf curling (Figure 4). Meanwhile, 8/10 plants inoculated with SLCV DNA-A and WCSV DNA-B were found to be co-infected. Both SLCV DNA-A and WCSV DNA-B were detected by PCR in newly emerged leaves, which indicates systemic infection. The plants showed mild symptoms of leaf curling when compared to the symptoms displayed by the plants inoculated with both SLCV components (Figure 4). This demonstrates that SLCV DNA-A is able to pseudo-recombine with WCSV DNA-B causing mild symptoms in *N. benthamiana* plants. However, the ability of this SLCV-DNA-A/WCSV-DNA-B combination to infect plants in the family Cucurbitaceae needs to be investigated since these would presumably be the natural hosts of such combinations.

On the other hand, infectivity assays with both components of WCSV did not yield any infections. None of the inoculated plants displayed symptoms and neither of the WCSV components were detected by PCR in any of the *N. benthamiana* plants inoculated at 15 dpi. It is plausible that the infection assay did not work efficiently; however, the components used for these experiments were isolated from different plants since none of the plants screened in this study had both components of WCSV (Table 1). No infection could be detected by PCR for the plants inoculated with WCSV DNA-A and SLCV DNA-B, but since the infection from WCSV components did not yield an infection, it is possible that the WCSV DNA-A clones used for these experiments were in some way defective.

**Figure 5:**
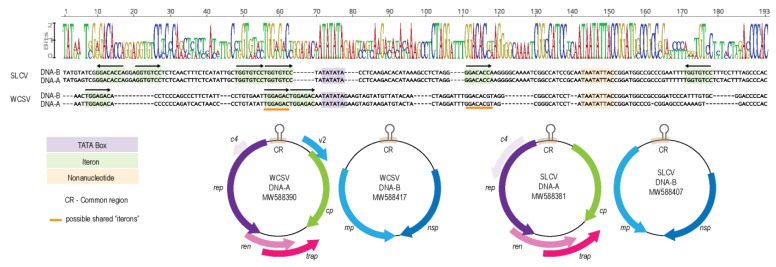
Alignment of the common region from SLCV and WCSV genomic components. Highlighted in purple is the TATA box, in light orange is the nonanucleotide sequence, in green are the identified “iteron” sequences for each begomovirus and the orange line indicate possible shared “iterons” between SLCV and WCSV. Graphical representation of the genomic organization of the DNA-A and DNA-B from SLCV and WCSV showing the position of the common region (CR) on the genome. *rep*—replication associated protein; *cp*—capsid protein; *ren*—replication enhancer protein; *trap*—transactivator protein; *v2*—movement proteins; *c4*—symptom determinant protein; *mp*—movement protein and *nsp*—nuclear shuttle protein.

The similarity of the common region (CR) between a pair of DNA-A and DNA-B components is cardinal for the capacity of DNA-A to trans-replicate DNA-B. Besides a conserved nonanucleotide sequence within a stem-loop structure at the origin of virion replication, the CR also contains TATA boxes and replication-associated iterative sequences called “iterons”. Iteron sequence similarity between DNA-A and DNA-B components is essential for effective trans-replication. The SLCV CR has TATA box and iteron sequences that are typical of WCSV isolates (TGGTGTCC) that occur as four repeats and two inverted repeats [52] (Figure 5). In comparison, the WCSV CR has the iteron sequence typical of Old World begomoviruses, containing three previously reported directly repeated “TGGAGAC” sequences upstream of the TATA box [19,20,53] (Figure 5).

Since the SLCV DNA-A is able to trans-replicate the WCSV DNA-B, it was expected that the iteron sequences of WCSV DNA-B would be similar to those of SLCV. However, the alignment of the CR of the SLCV and WCSV components show that there are only two regions flanking the TATA box in the WCSV CR that have any observable similarity to the SLCV iterons: an inverted repeat, “GGACACgt” and a direct repeat “TGGaGaCt” (Figure 5). Nevertheless, our inoculation assay results demonstrate that the Rep of SLCV DNA-A is able to bind to the CR of the WCSV DNA-B to initiate replication. Similarly, viable pseudo-recombination has also been detected for the DNA-A and DNA-B of two tomato-infecting begomoviruses with different “iteron” sequences [54]. In previous reports of successful pseudo-recombination between WCSV and tomato leaf curl Palampur virus (ToLCPMV; [51]) and between SLCV and cucurbit leaf crumple virus (CuLCV; [6] the viruses shared similar iterons. It is possible that the iteron sequences are not the only factor contributing to the successful trans-replication of the WCSV DNA-B.

## 4. Concluding Remarks

This study reports the detection of SLCV (24 DNA-A and 22 DNA-B) and WCSV (3 DNA-A and 3 DNA-B) in 23 cactus plants, as well as 10 other plants from the USA and two cactus plants from Mexico (Table 1). Both SLCV and WCSV were also identified in cultivated plants sampled in the state of Arizona (USA) showing those viruses are actively circulating in the cropping systems of the area. Interestingly, that diverse begomoviruses are associated with cactus may represent a spillover event from agricultural areas into native vegetation. A broad diversity of native Cucurbitaceae plants are also found in North America and could further act as intermediate reservoirs of SLCV and WCSV in agro-ecological areas. It is important to point out that some of the cactus plants from the USA originated from other countries but had been cultivated at the Desert Botanical Garden in Phoenix (USA) for years before being sampled for this study. Hence, it cannot be ruled out that those plants might have been infected before coming to the USA.

The identification of WCSV in the USA and Mexico suggests that this begomovirus may be broadly distributed in North America. Phylogenetic analysis suggests that WCSV may have been introduced to the New World on two different occasions. It is unclear whether WCSV, an Old World begomovirus, will have as severe an impact in North America as it has had in the Middle East [15,20,46]. Future surveys need to be conducted to determine the distribution and spread of the virus in North America, especially given that the insect vector *Bemisia tabaci* for begomoviruses is widespread on the continent. The Old World begomovirus tomato yellow leaf curl virus (TYLCV) is an example of a successful “invasive” species in the New World [55,56,57].

Here, we also demonstrate that SLCV DNA-A is able to replicate the WCSV DNA-B, causing mild leaf curling symptoms. Although the exact molecular mechanism for this is unclear, since these molecules do not share a similar common region or iteron sequences, this demonstrates that encounters between those two viruses in mixed infection can lead to pseudo-recombination in nature. Previous reports of pseudo-recombination for WCSV with tomato leaf curl Palampur virus [51] and SLCV with cucurbit leaf crumple virus [6] exist. However, in both cases, the viruses shared similar iterons. The fact that begomovirus components with no iteron similarity were able to pseudo-recombine brings into question what other molecular interactions are necessary for the initiation of replication.

Taken together, our results suggest that future surveillance studies are essential to determine the distribution of the SLCV and WCSV globally. Even though the cactus plants with begomovirus infection did not display any apparent symptoms, there is no information on any long-term threat from begomoviruses to the local cactus populations. Additionally, the vegetative propagation of several economically important cacti, such as *Opuntia ficus-indica* and *Selenicereus undatus*, may represent a disregarded means by which begomoviruses, as well as other geminiviruses that we have recently identified [27,58] in cactus plant, could be spread throughout the world.

## Figures and Tables

**Table 1:** Summary of the begomovirus components identified in each plant host, including the host family species, country and year of collection.

Begomovirus	Accession #	Host	Isolate	Region of Collection	Collection Year	Component
Squash leaf curl virus KPB1 SB	MW588396	*Capsicum annuum* Jalapeño	KPB1_SB	USA	2019	B
Squash leaf curl virus SF6	MW588388	*Capsicum* sp.	SF6_SA	USA	2019	A
	MW588413		SF6_SB	USA	2019	B
Squash leaf curl virus SF3 SA	MW588385	*Solanum melongena*	SF3_SA	USA	2019	A
Squash leaf curl virus SF4	MW588386	*Cucurbita pepo*	SF4_SA	USA	2019	A
	MW588411		SF4_SB	USA	2019	B
Squash leaf curl virus LCM95 SP41	MW588383	*Cylindropuntia whipplei*	LCM95_SP41	USA	2015	A
Squash leaf curl virus LCM93	MW588409	*Ferocactus cylindraceus*	LCM93_SP58	USA	2016	B
	MW588410		LCM93_SP2634	USA	2016	B
Squash leaf curl virus LCM90 SP344	MW588382	*Leptocereus quadricostatus*	LCM90_SP344	USA	2015	A
Squash leaf curl virus LCM67 SP209	MW588400	*Opuntia andersoni*	LCM67_SP209	Mexico	2009	B
Squash leaf curl virus LCM65	MW588372	*Opuntia arenaria*	LCM65_SP41	USA	2009	A
	MW588373		LCM65_SP344_1	USA	2009	A
	MW588374		LCM65_SP344_2	USA	2009	A
	MW588398		LCM65_SP209	USA	2009	B
Squash leaf curl virus LCM69	MW588375	*Opuntia atrispina*	LCM69_SP41	USA	2009	A
	MW588376		LCM69_SP341	USA	2009	A
	MW588377		LCM69_SP344	USA	2009	A
Squash leaf curl virus LCM66 SP209	MW588399	*Opuntia aureispina*	LCM66_SP209	USA	2006	B
Squash leaf curl virus LCM89	MW588381	*Opuntia basilaris* var. *longiareolata*	LCM89_SP41	USA	2015	A
	MW588407		LCM89_SP58	USA	2015	B
	MW588408		LCM89_SP2634	USA	2015	B
Squash leaf curl virus LCM68 SP209	MW588401	*Opuntia boldinghii*	LCM68_SP209	USA	2009	B
Squash leaf curl virus LCM74 SP1332	MW588402	*Opuntia bravoana*	LCM74_SP1332	Mexico	2009	B
Squash leaf curl virus LCM76	MW588380	*Opuntia caracassana*	LCM76_SA	USA	2009	A
	MW588404		LCM76_SP1332	USA	2009	B
Squash leaf curl virus LCM75	MW588378	*Opuntia carstenii*	LCM75_SP41	Mexico	2009	A
	MW588379		LCM75_SP344	Mexico	2009	A
	MW588403		LCM75_SP1332	Mexico	2009	B
Squash leaf curl virus LCM78 SP1332	MW588405	*Opuntia chaffeyi*	LCM78_SP1332	Mexico	2006	B
Squash leaf curl virus LCM79 SP1332	MW588406	*Opuntia chaffeyi*	LCM79_SP1332	Mexico	2006	B
Squash leaf curl virus LCM56 SP341	MW588369	*Opuntia guatemalensis*	LCM56_SP341	USA	2009	A
Squash leaf curl virus LCM58 SP41	MW588370	*Opuntia hondurensis*	LCM58_SP41	USA	2009	A
Squash leaf curl virus LCM60 SP341	MW588371	*Opuntia inaperta*	LCM60_SP341	USA	2009	A
Squash leaf curl virus LCM62 SP8848	MW588397	*Opuntia karwinskiana*	LCM62_SP8848	USA	2009	B
Squash leaf curl virus LCM29 SP41	MW588368	*Opuntia puberula*	LCM29_SP41	Mexico	2009	A
Squash leaf curl virus DBG34 SP15973	MW588395	*Opuntia robusta*	DBG34_SP15973	USA	2018	B
Squash leaf curl virus LCM96 SP41	MW588384	*Pereskiopsis kellermannii*	LCM96_SP41	USA	2016	A
Squash leaf curl virus SF5	MW588387	*Raphanus sativus*	SF5_SA	USA	2019	A
	MW588412		SF5_SB	USA	2019	B
Squash leaf curl virus CG5	MW588366	*Solanum lycopersicum*	CG5_SA	USA	2018	A
	MW588393		CG5_SB	USA	2018	B
Squash leaf curl virus CG6	MW588367	*Solanum lycopersicum*	CG6_SA	USA	2018	A
	MW588394		CG6_SB	USA	2018	B
Squash leaf curl virus SWAT	MW588389	*Citrullus lanatus*	SWAT_SA	USA	2019	A
	MW588414		SWAT_SB	USA	2019	B
Watermelon chlorotic stunt virus HERB15	MW588391	*Solanum* sp.	HERB15	USA	2019	A
Watermelon chlorotic stunt virus HERB28	MW588392	*Stachys byzantina*	HERB28	USA	2019	A
Watermelon chlorotic stunt virus LCM53	MW588417	*Opuntia cochenillifera*	LCM53	USA	2006	B
Watermelon chlorotic stunt virus LCM 52	MW588390	*Opuntia auberi*	LCM_52	Mexico	2006	A
Watermelon chlorotic stunt virus LCM51	MW588416	*Opuntia auberi*	LCM51	Mexico	2006	B
Watermelon chlorotic stunt virus LCM50	MW588415	*Consolea spinosissima*	LCM50	USA	2006	B

## Data Availability

Sequence determined as part of this study have been deposited in GenBank under accession #s MW588366-MW588417.

## Supplementary Materials

The following are available online at https://www.mdpi.com/article/10.3390/v13050810/s1, Supplementary Data S1: Genomewide pairwise identity comparison of the SLCV DNA-A and DNA-B sequences from this study together with SLCV sequences in GenBank; Supplementary Data S2. Genomewide pairwise identity comparison of the WCSV DNA-A and DNA-B sequences in this study together with WCSV sequences in GenBank; Supplementary Table S1: Details of the noncactus plants sampled as part of this study including plant species, family, collection location, country and date; Supplementary Table S2: Pairs of abutting primers used in this study.

## References

[1] Zhang W., Olson N.H., Baker T.S., Faulkner L., Agbandje-McKenna M., Boulton M.I., Davies J.W., McKenna R. (2001). Structure of the Maize streak virus geminate particle. Virology.

[2] Navas-Castillo J., Fiallo-Olivé E., Sánchez-Campos S. (2011). Emerging virus diseases transmitted by whiteflies. Annu. Rev. Phytopathol..

[3] Rojas M.R., Macedo M.A., Maliano M.R., Soto-Aguilar M., Souza J.O., Briddon R.W., Kenyon L., Rivera Bustamante R.F., Zerbini F.M., Adkins S. (2018). World Management of Geminiviruses. Annu. Rev. Phytopathol..

[4] Lazarowitz S.G. (1991). Molecular characterization of two bipartite geminiviruses causing squash leaf curl disease: Role of viral replication and movement functions in determining host range. Virology.

[5] Flock R.A., Mayhew D.E. (1981). Squash leaf curl, a new disease of cucurbits in California. Plant Dis..

[6] Brown J.K., Idris A.M., Alteri C., Stenger D.C. (2002). Emergence of a New Cucurbit-Infecting Begomovirus Species Capable of Forming Viable Reassortants with Related Viruses in the Squash leaf curl virus Cluster. Phytopathology.

[7] Kuo Y.W., Rojas M.R., Gilbertson R.L., Wintermantel W.M. (2007). First Report of Cucurbit yellow stunting disorder virus in California and Arizona, in Association with Cucurbit leaf crumple virus and Squash leaf curl virus. Plant Dis..

[8] Medina-Hernández D., Caamal-Chan M.G., Vargas-Salinas M., Loera-Muro A., Barraza A., Holguín-Peña R.J. (2019). Molecular characterization and phylogenetic analysis of a Squash leaf curl virus isolate from Baja California Sur, Mexico. PeerJ.

[9] Antignus Y., Lachman O., Pearlsman M., Omer S., Yunis H., Messika Y., Uko O., Koren A. (2003). Squash leaf curl geminivirus—A new illegal immigrant from the Western Hemisphere and a threat to cucurbit crops in Israel. Phytoparasitica.

[10] Al-Musa A., Anfoka G., Misbeh S., Abhary M., Ahmad F.H. (2008). Detection and Molecular Characterization of Squash leaf curl virus (SLCV) in Jordan: Molecular Characterization of Squash leaf curl virus. J. Phytopathol..

[11] Idris A.M., Abdel-Salam A., Brown J.K. (2006). Introduction of the New World Squash leaf curl virus to Squash (*Cucurbita pepo*) in Egypt: A Potential Threat to Important Food Crops. Plant Dis..

[12] Shahid M.S., Al-Sulaimani H., Al-Sadi A.M. (2020). Squash Leaf Curl Virus: A New World Bipartite Begomovirus Threatening Squash Production in Oman. Plant Dis..

[13] Sobh H., Samsatly J., Jawhari M., Najjar C., Haidar A., Abou-Jawdah Y. (2012). First Report of Squash leaf curl virus in Cucurbits in Lebanon. Plant Dis..

[14] Ali-Shtayeh M.S., Jamous R.M., Husein E.Y., Alkhader M.Y. (2010). First Report of Squash leaf curl virus in Squash (*Cucurbita pepo*), Melon (*Cucumis melo*), and Cucumber (*Cucumis sativa*) in the Northern West Bank of the Palestinian Authority. Plant Disease.

[15] Abudy A., Sufrin-Ringwald T., Dayan-Glick C., Guenoune-Gelbart D., Livneh O., Zaccai M., Lapidot M. (2010). Watermelon chlorotic stunt and Squash leaf curl begomoviruses—New threats to cucurbit crops in the Middle East. Isr. J. Plant Sci..

[16] Ahmad F.H., Odeh W., Anfoka G. (2013). First Report on the Association of Squash leaf curl virus and Watermelon chlorotic stunt virus with Tomato Yellow Leaf Curl Disease. Plant Dis..

[17] Sufrin-Ringwald T., Lapidot M. (2011). Characterization of a Synergistic Interaction Between Two Cucurbit-Infecting Begomoviruses: Squash leaf curl virus and Watermelon chlorotic stunt virus. Phytopathology.

[18] Bedford I.D., Briddon R.W., Jones P., Alkaff N., Markham P.G. (1994). Differentiation of three whitefly-transmitted geminiviruses from the Republic of Yemen. Eur. J. Plant Pathol..

[19] Al-Musa A., Anfoka G., Al-Abdulat A., Misbeh S., Haj Ahmed F., Otri I. (2011). Watermelon chlorotic stunt virus (WmCSV): A serious disease threatening watermelon production in Jordan. Virus Genes.

[20] Ali-Shtayeh M.S., Jamous R.M., Mallah O.B., Abu-Zeitoun S.Y. (2014). Molecular characterization of watermelon chlorotic stunt virus (WmCSV) from Palestine. Viruses.

[21] Khan A.J., Akhtar S., Briddon R.W., Ammara U., Al-Matrooshi A.M., Mansoor S. (2012). Complete nucleotide sequence of watermelon chlorotic stunt virus originating from Oman. Viruses.

[22] Kheyr-Pour A., Bananej K., Dafalla G.A., Caciagli P., Noris E., Ahoonmanesh A., Lecoq H., Gronenborn B. (2000). Watermelon chlorotic stunt virus from the Sudan and Iran: Sequence Comparisons and Identification of a Whitefly-Transmission Determinant. Phytopathology.

[23] Rezk A.A., Sattar M.N., Alhudaib K.A., Soliman A.M. (2019). Identification of watermelon chlorotic stunt virus from watermelon and zucchini in Saudi Arabia. Can. J. Plant Pathol..

[24] Bananej K., Ahoonmanesh A., Kheyr-Pour A. (2002). Host Range of an Iranian Isolate of Watermelon Chlorotic Stunt Virus as Determined by Whitefly-mediated Inoculation and Agroinfection, and its Geographical Distribution. J. Phytopathol..

[25] Abou-Jawdah Y., Sobh H., Haidar A., Samsatly J., Barba M., Motta E., Tomassoli L., Riccioni L. (2010). First report in Lebanon on detection of two whitefly transmitted cucurbit viruses and their molecular characterization. Petria, Giornale di Patologia delle Piante 20, Proceedings of the 13th Congress of the Mediterranean Phytopathological Union, MPU, Plant Pathology Research Centre in Rome, Rome, Italy, 20–25 June 2010.

[26] Domínguez-Durán G., Rodríguez-Negrete E.A., Morales-Aguilar J.J., Camacho-Beltrán E., Romero-Romero J.L., Rivera-Acosta M.A., Leyva-López N.E., Arroyo-Becerra A., Méndez-Lozano J. (2018). Molecular and biological characterization of Watermelon chlorotic stunt virus (WmCSV): An Eastern Hemisphere begomovirus introduced in the Western Hemisphere. Crop Prot..

[27] Fontenele R.S., Salywon A.M., Majure L.C., Cobb I.N., Bhaskara A., Avalos-Calleros J.A., Argüello-Astorga G.R., Schmidlin K., Khalifeh A., Smith K. (2020). A Novel Divergent Geminivirus Identified in Asymptomatic New World Cactaceae Plants. Viruses.

[28] Rojas M.R. (1993). Use of degenerate primers in the polymerase chain reaction to detect Whitefly-transmitted geminiviruses. Plant Dis..

[29] Lindbo J.A. (2007). TRBO: A high-efficiency tobacco mosaic virus RNA-based overexpression vector. Plant Physiol..

[30] Ferro M.M.M., Ramos-Sobrinho R., Xavier C.A.D., Zerbini F.M., Lima G.S.A., Nagata T., Assunção I.P. (2019). New approach for the construction of infectious clones of a circular DNA plant virus using Gibson Assembly. J. Virol. Methods.

[31] Gibson D.G., Young L., Chuang R.-Y., Venter J.C., Hutchison C.A., Smith H.O. (2009). Enzymatic assembly of DNA molecules up to several hundred kilobases. Nat. Methods.

[32] Katoh K., Standley D.M. (2013). MAFFT multiple sequence alignment software version 7: Improvements in performance and usability. Mol. Biol. Evol..

[33] Martin D.P., Varsani A., Roumagnac P., Botha G., Maslamoney S., Schwab T., Kelz Z., Kumar V., Murrell B. (2020). RDP5: A computer program for analysing recombination in, and removing signals of recombination from, nucleotide sequence datasets. Virus Evol..

[34] Martin D., Rybicki E. (2000). RDP: Detection of recombination amongst aligned sequences. Bioinformatics.

[35] Padidam M., Sawyer S., Fauquet C.M. (1999). Possible emergence of new geminiviruses by frequent recombination. Virology.

[36] Martin D.P., Posada D., Crandall K.A., Williamson C. (2005). A modified bootscan algorithm for automated identification of recombinant sequences and recombination breakpoints. AIDS Res. Hum. Retrovir..

[37] Smith J.M. (1992). Analyzing the mosaic structure of genes. J. Mol. Evol..

[38] Posada D., Crandall K.A. (2001). Evaluation of methods for detecting recombination from DNA sequences: Computer simulations. Proc. Natl. Acad. Sci. USA.

[39] Gibbs M.J., Armstrong J.S., Gibbs A.J. (2000). Sister-scanning: A Monte Carlo procedure for assessing signals in recombinant sequences. Bioinformatics.

[40] Boni M.F., Posada D., Feldman M.W. (2007). An exact nonparametric method for inferring mosaic structure in sequence triplets. Genetics.

[41] Kalyaanamoorthy S., Minh B.Q., Wong T.K.F., von Haeseler A., Jermiin L.S. (2017). ModelFinder: Fast model selection for accurate phylogenetic estimates. Nat. Methods.

[42] Nguyen L.-T., Schmidt H.A., von Haeseler A., Minh B.Q. (2015). IQ-TREE: A fast and effective stochastic algorithm for estimating maximum-likelihood phylogenies. Mol. Biol. Evol..

[43] Stöver B.C., Müller K.F. (2010). TreeGraph 2: Combining and visualizing evidence from different phylogenetic analyses. BMC Bioinform..

[44] Letunic I., Bork P. (2019). Interactive Tree Of Life (iTOL) v4: Recent updates and new developments. Nucleic Acids Res..

[45] Muhire B.M., Varsani A., Martin D.P. (2014). SDT: A virus classification tool based on pairwise sequence alignment and identity calculation. PLoS ONE.

[46] Lapidot M., Gelbart D., Gal-On A., Sela N., Anfoka G., Haj Ahmed F., Abou-Jawada Y., Sobh H., Mazyad H., Aboul-Ata A.-A.E. (2014). Frequent migration of introduced cucurbit-infecting begomoviruses among Middle Eastern countries. Virol. J..

[47] Brown J., Baumann K., Idris A. (2005). Characterization of squash leaf curl and squash mild leaf curl viruses: Host range and reassortment for four SLCV clade viruses. Phytopathology.

[48] Briddon R.W., Patil B.L., Bagewadi B., Nawaz-ul-Rehman M.S., Fauquet C.M. (2010). Distinct evolutionary histories of the DNA-A and DNA-B components of bipartite begomoviruses. BMC Evol. Biol..

[49] De Bruyn A., Villemot J., Lefeuvre P., Villar E., Hoareau M., Harimalala M., Abdoul-Karime A.L., Abdou-Chakour C., Reynaud B., Harkins G.W. (2012). East African cassava mosaic-like viruses from Africa to Indian ocean islands: Molecular diversity, evolutionary history and geographical dissemination of a bipartite begomovirus. BMC Evol. Biol..

[50] Mohammed H.S., El Siddig M.A., El Hussein A.A., Navas-Castillo J., Fiallo-Olivé E. (2017). First Report of Datura innoxia as a Natural Host of Watermelon chlorotic stunt virus in Sudan. Plant Dis..

[51] Esmaeili M., Heydarnejad J., Massumi H., Varsani A. (2015). Analysis of watermelon chlorotic stunt virus and tomato leaf curl Palampur virus mixed and pseudo-recombination infections. Virus Genes.

[52] Argüello-Astorga G.R., Guevara-González R.G., Herrera-Estrella L.R., Rivera-Bustamante R.F. (1994). Geminivirus replication origins have a group-specific organization of iterative elements: A model for replication. Virology.

[53] Al-Saleh M.A., Ahmad M.H., Al-Shahwan I.M., Brown J.K., Idris A.M. (2014). First Report of Watermelon chlorotic stunt virus Infecting Watermelon in Saudi Arabia. Plant Dis..

[54] Galvão R.M., Mariano A.C., Luz D.F., Alfenas P.F., Andrade E.C., Zerbini F.M., Almeida M.R., Fontes E.P.B. (2003). A naturally occurring recombinant DNA-A of a typical bipartite begomovirus does not require the cognate DNA-B to infect Nicotiana benthamiana systemically. J. Gen. Virol..

[55] Duffy S., Holmes E.C. (2007). Multiple introductions of the Old World begomovirus Tomato yellow leaf curl virus into the New World. Appl. Environ. Microbiol..

[56] Mabvakure B., Martin D.P., Kraberger S., Cloete L., van Brunschot S., Geering A.D.W., Thomas J.E., Bananej K., Lett J.-M., Lefeuvre P. (2016). Ongoing geographical spread of Tomato yellow leaf curl virus. Virology.

[57] Lefeuvre P., Martin D.P., Harkins G., Lemey P., Gray A.J.A., Meredith S., Lakay F., Monjane A., Lett J.-M., Varsani A. (2010). The spread of tomato yellow leaf curl virus from the Middle East to the world. PLoS Pathog..

[58] Fontenele R.S., Salywon A.M., Majure L.C., Cobb I.N., Bhaskara A., Avalos-Calleros J.A., Arguello-Astorga G.R., Schmidlin K., Khalifeh A., Smith K. (2021). New World Cactaceae plants harbor diverse geminiviruses. Viruses.

