# Supplementary material for: Identification of the Begomoviruses Squash Leaf Curl Virus and Watermelon Chlorotic Stunt Virus in Various Plant Samples in North America
Source: Viruses. 2021 Apr 30;13(5):810. doi: 10.3390/v13050810 (PMC8147125; doi:10.3390/v13050810)
Supplement: Supplementary file 1 [file viruses-13-00810-s001.zip › Supplementary_Table.pdf]

Supplementary Table S1

Sample #	Lab ID	Sample	Country	Collection year	Note	Family
1	DBGHerb1	*Amaranthus* sp.	USA	2019	Desert botanical garden	Amaranthaceae
2	DBGHerb2	*Helianthus* sp.	USA	2019	Desert botanical garden	Asteraceae
3	DBGHerb3	*Cucurbita* sp.	USA	2019	Desert botanical garden	Cucurbitaceae
4	DBGHerb4	*Capsicum* sp.	USA	2019	Desert botanical garden	Solanaceae
5	DBGHerb5	*Capsicum* sp.	USA	2019	Desert botanical garden	Solanaceae
6	DBGHerb6	*Cucurbita* sp.	USA	2019	Desert botanical garden	Cucurbitaceae
7	DBGHerb7	*Albemochus esculenta*	USA	2019	Desert botanical garden	Malvaceae
8	DBGHerb8	*Solanum* sp.	USA	2019	Desert botanical garden	Solanaceae
9	DBGHerb9	*Solanum* sp.	USA	2019	Desert botanical garden	Solanaceae
10	DBGHerb10	*Ocimum basilicum*	USA	2019	Desert botanical garden	Lamiaceae
11	DBGHerb11	*Solanum* sp.	USA	2019	Desert botanical garden	Solanaceae
12	DBGHerb12	*Cucurbita* sp.	USA	2019	Desert botanical garden	Cucurbitaceae
13	DBGHerb13	*Apium* sp.	USA	2019	Desert botanical garden	Apiaceae
14	DBGHerb14	*Allium* sp.	USA	2019	Desert botanical garden	Alliaceae
15	DBGHerb15	*Solanum* sp.	USA	2019	Desert botanical garden	Solanaceae
16	DBGHerb16	*Cynara scolymus*	USA	2019	Desert botanical garden	Asteraceae
17	DBGHerb17	*Jasminum* sp.	USA	2019	Desert botanical garden	Oleaceae
18	DBGHerb19	*Laurus nobilis*	USA	2019	Desert botanical garden	Lauraceae
19	DBGHerb20	*Rosmarinus officinalis*	USA	2019	Desert botanical garden	Lamiaceae
20	DBGHerb21	*Ocimum* sp.	USA	2019	Desert botanical garden	Lamiaceae
21	DBGHerb22	*Cucumis melo*	USA	2019	Desert botanical garden	Cucurbitaceae
22	DBGHerb23	*Solanum melongena*	USA	2019	Desert botanical garden	Solanaceae
23	DBGHerb24	*Stevia* sp.	USA	2019	Desert botanical garden	Asteraceae
24	DBGHerb28	*Stachys byzantina*	USA	2019	Desert botanical garden	Lamiaceae
25	DBGHerb29	*Apium graveolens*	USA	2019	Desert botanical garden	Apiaceae
26	KPB1	*Capsicum annuum* 'Jalapeño'	USA	2019	Mountain View Park	Solanaceae
27	KPB2	*Solanum lycopersicum* var. *cerasiforme*	USA	2019	Mountain View Park	Solanaceae
28	KPB3	Basil	USA	2019	Mountain View Park	Lamiaceae
29	KPB4	Unknown	USA	2019	Mountain View Park	Unknown
30	SF1	Unknown	USA	2019	Schnepf Farms	Unknown
31	SF2	*Raphanus sativus*	USA	2019	Schnepf Farms	Brassicaceae
32	SF3	*Solanum melongena*	USA	2019	Schnepf Farms	Solanaceae
33	SF4	*Cucurbita* sp.	USA	2019	Schnepf Farms	Cucurbitaceae
34	SF5	unknown	USA	2019	Schnepf Farms	unknown
35	SF6	*Capsicum* sp.	USA	2019	Schnepf Farms	Solanaceae
36	MVN1	*Physalis philadelphica*	USA	2019	Moon Valley Nursery	Solanaceae
37	MVN2	*Solanum lycopersicum*	USA	2019	Moon Valley Nursery	Solanaceae
38	MVN3	*Solanum lycopersicum*	USA	2019	Moon Valley Nursery	Solanaceae
39	MVN4	*Solanum lycopersicum*	USA	2019	Moon Valley Nursery	Solanaceae
40	MVN5	*Solanum lycopersicum* 'Early Girl'	USA	2019	Moon Valley Nursery	Solanaceae
41	MVN6	*Solanum lycopersicum* 'Better Boy'	USA	2019	Moon Valley Nursery	Solanaceae
42	MVN7	*Solanum lycopersicum* var. cerasiforme	USA	2019	Moon Valley Nursery	Solanaceae
43	MVN8	*Solanum lycopersicum* 'Celebrity'	USA	2019	Moon Valley Nursery	Solanaceae
44	MVN9	*Solanum lycopersicum*	USA	2019	Moon Valley Nursery	Solanaceae
45	MVN10	*Solanum lycopersicum*	USA	2019	Moon Valley Nursery	Solanaceae
46	CG5	*Solanum lycopersicum*	USA	2018	Community garden	Solanaceae
47	CG6	*Solanum lycopersicum*	USA	2018	Community garden	Solanaceae
48	SWAT	*Citrullus lanatus*	USA	2018	Community garden	Cucurbitaceae

Supplementary Table S2

Virus target	Primer	Sequence
SLCV DNA-A	LCM_SP_41_F	AGCTGACATATCCCATACCTCTTTAATTCA
SLCV DNA-A	LCM_SP_41_R	TTATTTATAGGGACCACACTTAGTCACCAA
SLCV DNA-B	DBG_SP_15973_F	CTGTCGCTCATTATGTTTTGGAGAGTATTC
SLCV DNA-B	DBG_SP_15973_R	CATTAACAACCATTGTGAGTGTTAAACGAA
SLCV DNA-B	LCM_SP_1332_F	TGCGATTGGGAAAAGTCTTAACATAACTAG
SLCV DNA-B	LCM_SP_1332_R	CAAGAACGTACATAAAATTGAAACGACTGC
SLCV DNA-B	LCM_SP_2634_F	ATGTAATTGGGATTAGCTTAGGGATTTTGG
SLCV DNA-B	LCM_SP_2634_R	TATTAGTTCGCTTTACGCTCACATTAGTTG
SLCV DNA-B	LCM_SP_58_F	TTAACTTCGCCTTTAATTTGTACTGTCACA
SLCV DNA-B	LCM_SP_58_R	TCACTTTGTCAATTTGAATTAAAGTCGAGC
WCSV DNA-A	WCSV_A_F	TTCTAAATACCCTTAAGAAACGACCAGTCT
WCSV DNA-A	WCSV_A_R	CGCAGGTTCTAAAATACCTCGATATGTTAG
WCSV DNA-B	LCM_SP_242_F	AATCGTATTGGGTGTTTTGGAGTATTTCAT
WCSV DNA-B	LCMC_SP_242_R	ACCAGCTCAATTACTTTCGCTTAATTATCC
